# Exosome-Derived Noncoding RNAs as a Promising Treatment of Bone Regeneration

**DOI:** 10.1155/2021/6696894

**Published:** 2021-01-21

**Authors:** Bei Yin, Qingge Ma, Chenghao Song, Lingyi Zhao, Fanyuan Yu, Chenglin Wang, Yu Shi, Ling Ye

**Affiliations:** ^1^State Key Laboratory of Oral Diseases & National Clinical Research Center for Oral Diseases, West China Hospital of Stomatology, Sichuan University, Chengdu, China; ^2^Department of Endodontics, West China Hospital of Stomatology, Sichuan University, Chengdu, China

## Abstract

The reconstruction of large bone defects remains a crucial challenge in orthopedic surgery. The current treatments including autologous and allogenic bone grafting and bioactive materials have their respective drawbacks. While mesenchymal stem cell (MSC) therapy may address these limitations, growing researches have demonstrated that the effectiveness of MSC therapy depends on paracrine factors, particularly exosomes. This aroused great focus on the exosome-based cell-free therapy in the treatment of bone defects. Exosomes can transfer various cargoes, and noncoding RNAs are the most widely studied cargo through which exosomes exert their ability of osteoinduction. Here, we review the research status of the exosome-derived noncoding RNAs in bone regeneration, the potential application of exosomes, and the existing challenges.

## 1. Current Status of Bone Regeneration

The reconstruction of large bone defects is a key challenge in reconstructive surgery. Currently, the treatment strategies include autologous and allogenic bone grafting, bioactive materials. However, they all have their limitations in various aspects. Autologous bone grafts have the disadvantages of limited sources of bone and extra surgical injuries. Allogenic bone grafts may cause immunological rejection and disease transmission. As for the bioactive materials, the issues of biocompatibility, structural stability, mechanical strength, and degradability remain to be solved.

Stem cell-based engineered bone may address the limitations of the abovementioned methods. Nevertheless, the satisfactory results are hindered by MSCs' limited ability to form enough new bone. Therefore, approaches of enhancing MSC's osteogenic differentiation have been investigated such as gene editing [1, 2], the use of growth factors [3], and the addition of cell-derived conditioned medium (CM) [4, 5]. However, several considerations need to be clarified in terms of cell transplantation. For example, genetic modification gives rise to safety issues; the concentration of biotherapeutics in CM is low, and CM may contain medium contaminants. Moreover, stem cell therapy is further hindered by insufficient cell number, complex and costly expansion procedures, immunological rejection, the accumulation of genomic alterations [6], the risk of tumor [7], the formation of emboli [8], and so forth.

## 2. Exosomes

In recent years, accumulating researches demonstrated that the paracrine role may account for the efficacy of MSC therapy given that limited cells have engrafted into the sites of injury [9, 10] and that bone regeneration can be regulated by paracrine factors [11, 12]. In this scenario, exosome transplantation is considered as a novel cell-free therapy for bone regeneration. Exosomes are 40–100 nm extracellular vesicles (EVs). They are released by multivesicular bodies (MVBs) after fusion with cytomembranes. MVBs are late endosomes with many intraluminal vesicles inside formed by inward budding of endosomal membranes [13]. Exosomes can be transported to distant sites via body fluids, or they can be invaginated by residing cells. They exist in a variate of body fluids such as breast milk, saliva, lymph, and bile.

Unlike stem cell therapy, the application of exosomes involves fewer safety considerations. In fact, several clinical studies have proved the safety of exosomes in the treatment of cancers [14]. Several other registered NIH clinical trials for the treatment of ulcers, diabetes, and oral mucositis are undergoing. To date, MSCs are the most prolific producer of exosomes. Immortalization of MSCs has no effects on the yield or the properties of exosomes, while it compromises the differentiation potential of MSCs [15]. Moreover, exosomes can be engineered to act as the carriers of RNA-related products such as siRNA and shRNA, with enhanced efficacy compared to nanoparticles and liposomes [16, 17]. Because exosomes contain several transmembrane proteins which can promote endocytosis while prevent phagocytosis by monocytes [18]. Furthermore, exosomes have a high degree of stability, in that their potency can be maintained at −20°C for 6 months [19].

## 3. Exosomes Regulate Osteogenic Ability of MSCs

Exosomes can promote the osteogenic ability of MSCs, and the effects of exosomes often increase with increasing concentrations. Indeed, some studies reported that exosomes even outperformed the currently used osteoinductive cocktail, the conditioned medium, and the extracellular matrix (ECM) [20]. *In vivo* experiments also revealed that exosomes dramatically stimulated osteogenesis in calvarial defects [21], bone fracture [22], and radiation-induced bone loss [23]. Exosomes affect the osteogenic differentiation of recipient cells by regulating various signaling pathways including TGF-*β*1 pathway, Wnt/*β*-catenin pathway [24], and MAPK pathway [25] and by upregulating mRNA and protein expression of osteogenesis-related genes such as Runt-related transcription factor 2 (RUNX2), Osteocalcin (OC), and Osterix (OSX). While exosomes derived from osteogenic conditions performed better in terms of bone regeneration [26], it is worth noting that exosomes from pathological MSCs such as MSCs from type 1 diabetes and those from the aged even inhibit the osteogenesis [27, 28].

Exosomes exert their influence either through interacting with the extracellular matrix or through internalization into cells. Once released, exosomes can anchor to the ECM and act as the initial sites for mineralized nodule along with matrix vesicles [29]. Recently, increasing researches have focused on the role of exosomes in delivering cargoes by internalization into cells. The various cargoes include proteins, nucleic acid, and lipid. Among them, noncoding RNAs (ncRNAs) are the most widely studied cargoes through which exosomes exert their ability of osteoinduction (Table 1).

## 4. Noncoding RNAs

High-throughput technologies have discovered about 90% of the genome is actively transcribed [30], but the majority (98%) of transcripts exist as ncRNAs. NcRNAs were initially regarded as transcriptional noise, but recent studies found they could exert regulative effects on various biological processes [31]. NcRNAs are divided into two classes based on the size, long ncRNAs (>200 nucleotides, long intergenic ncRNAs, antisense RNAs, etc.) and small ncRNAs (<200 nucleotides, including small interfering RNAs (siRNA), microRNAs (miRNAs), etc.).

### 4.1. MicroRNAs

miRNAs affect the expression of mRNAs by two modes including translational repression and mRNA decay, both of which were realized by the RNA-induced silencing complex (RISC) formed by miRNAs and Argonaute protein (Ago) [32]. When the miRNAs complement perfectly with the 3′ (or 5′ in some cases [33])—untranslated region (3′UTR) of the mRNAs, mRNA decay occurs through endonucleolytic cleavage by RISC. In the cases of partial complementation, RISC can recruit cofactor proteins to induce mRNA decay or translational repression in a manner independent of endonucleolytic cleavage [34, 35]. The MSC exosomal miRNAs are enriched in various KEGG pathways: Wnt, MAPK, and PI3K-Akt may be the signaling pathways through which exosomal miRNAs exert their effects [36]. Pathways including endocytosis and actin cytoskeleton are possibly related to the internalization of exosomes. Other pathways such as spliceosome, mRNA surveillance, and RNA transport are possible mechanisms of how miRNAs regulate the target cells [37].

The osteogenic induction of MSCs alters the expression of exosomal miRNAs. Several well-known suppressors of osteogenesis, such as miR-144, miR-31, and miR-221, were downregulated in exosomes from osteogenic differentiated MSCs, while positive regulators of osteogenesis like miR-21 were upregulated [38]. miR-31 and miR-221 can suppress osteogenic differentiation through targeting the 3′ untranslated regions of Runx2 and inhibiting Runx2 gene expression [39, 40]. miR-144-3p can target DNA demethylase ten-eleven translocation-2 (TET2), leading to the increase of 5-hydroxymethyl-cytosine (5hmC) levels and decrease of osteogenic genes expression [41]. Connexin-43 and Smad4 can also be targeted by miR-144-3p [42, 43]. miR-21 enhances osteogenic differentiation by downregulating Sox2 and Smad7 [44, 45].

Exosomal miRNA played an indispensable role in the cross-talk between bone and muscle. Myoblast-derived exosomes could deliver miR-27a-3p to the preosteoblasts and decrease the expression of adenomatous polyposis coli (APC), a negative regulator of *β*-catenin, thus, activating the *β*-catenin pathway. The effects of exosomes largely relied on miR-27a-3p, given that myoblast exosomes whose miR-27a-3p was inactivated lost their osteogenic-inductive capacity [46]. miR-27a-3p may also regulate osteogenic differentiation through targeting activating transcription factor 3 (ATF3). ATF3 can bind to the promoter of ALP and negatively regulate ALP expression [47].

Preosteoblast-derived exosomes contain miRNAs that can regulate the osteogenic differentiation. The preosteoblast exosomes contain abundant let-7 miRNA [48], which is a pivotal regulator of osteogenesis by targeting the high-mobility group AT-hook 2 (HMGA2) [49]. Exosomes from MC3T3-E1 cells facilitated the osteogenesis of bone marrow stromal ST2 cells. Through a study of the miRNA profile in ST2 cells and that in MC3T3-E1 exosomes, they identified several miRNAs (miR-7668-3p, miR-667-3p, miR-7044-5p, and miR-874-3p) that were transferred from preosteoblast exosomes to bone marrow stromal cells [50]. All the above miRNAs target Axin1, a suppressor of the Wnt signaling pathway, to exert their osteoinductive effects [51, 52].

In aging mice, the miRNA profile of exosomes was quite different from those in young mice. Specifically, the miR-183 cluster is enriched in aged exosomes [27]. miR-183-5p could increase the expression of the senescence marker *β*-galactosidase and suppress the osteogenic differentiation of BMSCs. Heme oxygenase-1 (Hmox1) is also a target of miR-183-5p, which has been shown previously to stimulate BMSCs osteogenic differentiation [53].

Apart from transporting microRNAs directly, exosomes can also transport protein to affect miRNA expression indirectly in recipient cells. In systemic lupus erythematosus (SLE) model Fas-deficient-MRL/lpr mice, exosome transplantation rescued the osteoporotic phenotype by the Fas/miR-29b/Dnmt1/Notch cascade. Exosome infusion provided donor-derived Fas to recipient cells, which facilitated the release of miR-29b into the extracellular environment and the decrease of intracellular miR-29b [54]. miR-29b decrease led to the upregulation of its direct target, DNA methyltransferase 1 (Dnmt1). Then, Dnmt1 controlled the hypermethylation and thus inactivation of Notch1, which is a negative regulator of osteogenesis [55].

### 4.2. Antisense lncRNAs

Antisense (AS) lncRNAs have sequences complementary to their sense counterparts. Antisense lncRNAs mainly function through regulating the expression of their sense transcripts. In some cases, they can form an RNA–RNA duplex with their sense counterparts, stabilizing their sense transcripts and thus increasing the gene expression [56, 57]. In other cases, AS lncRNAs mediate transcriptional repression of their sense protein-coding genes [58].

Exosome-derived antisense lncRNAs play a role in affecting the osteogenic ability of MSCs. Multiple myeloma (MM) is a plasma cell cancer characterized by multiple osteolytic damage. *In vitro* experiments demonstrated myeloma-derived exosomes could decrease MSCs' osteogenic differentiation ability. *In vivo* treatment of GW4869, an inhibitor of exosome secretion, attenuated bone loss in multiple myeloma models, with the expression of bone resorption marker beta-isomerized C-telopeptide (*β*-CTX) decreased and that of osteogenesis-related gene procollagen type I N-terminal propeptide (P1NP) increased. Using a lncRNA sequencing, they identified that antisense lncRNA RUNX-AS1 was enriched in MM-MSCs and MM-MSC-derived exosomes. Mechanismly, antisense lncRNA RUNX-AS1 and RUNX2 formed a RNA duplex at overlapping regions through base pairing, interfered with RUNX2 pre-mRNA splicing, and suppressed RUNX2 mRNA expression. Thereby, the MM-MSC-derived exosomes may transmit the antisense lncRNA RUNX-AS1 to MSCs, contributing to the impaired osteogenic differentiation ability in MM-MSCs [59].

### 4.3. Transfer RNA (tRNA) Halves

Mature cytoplasmic tRNAs can be cleavaged into small RNA fragments: the 5′ and 3′ tRNA halves (30–40 nt in size). The 5′ tRNA halves can silence target mRNAs by complementary base pairing to the 3′ UTRs of protein-coding genes in a manner like miRNA/siRNA [60, 61]. While previous studies found the 5′ tRNA halves were induced under stress to suppress translation and preserve energy, recent studies found that tRNA halves existed in certain types of cells persistently. Under physiological conditions, the bone marrow is the specific tissue expressing significant quantities of 5′ tRNA halves [62], while their level is quite low in several other tissues. MSC exosomes are abundant with 5′ tRNA halves with the targets of osteogenesis-related genes, such as RUNX2 and SMAD3 [63].

## 5. Strategies for Clinical Application of Exosomes

When it comes to clinical application, the pharmacokinetics of exosomes should be noted. One study showed that the exosomes predominately existed in the bone and lung 24 hours after injection [64]. However, other studies showed that the majority of exosomes were distributed in organs of rich vascular such as kidney, spleen, and lung [65, 66]. Contrary to systemic administration, local administration can maintain high concentrations of exosomes at target sites. Additionally, exosomes can be anchored to biomaterials/scaffolds, such as fibronectin, type I collagen, hydrogel, tricalcium phosphate, poly-lactic-glycolic acid (PLGA), and hydrogel glue, to support their delivery and to facilitate a controlled release of exosomes while enhancing the osteogenic ability of the biomaterials [67, 68]. It is worth noting that various aspects of the scaffolds could make influences on the behavior of the combined cells and the exosomes. For example, surface roughness plays a role in regulating both the mechanical strength of the material [69] and cell behavior. Whether the surface of the scaffolds affects the function of exosomes needs further study. To avoid the possible effects made by the heterogeneous morphology of the scaffolds when evaluating biomaterials carried with exosomes, the computer-aided design (CAD) technology may help implement a standardization of the shape and the surface of the scaffolds [70].

The osteogenic capacities of exosomes can be improved by modifying either the parent cells or the exosomes. The modifications include biochemical factors and mechanical factors. In fact, mechanical stimuli such as low-intensity pulsed ultrasound (LIPUS) could be used to enhance the osteoinductive capability of MSCs [71]. Whether the exosomes from the MSCs under such mechanical stimuli played a better role in bone regeneration needs further study. As for the biochemical factors, one study modified parent cells by miR-375-overexpressing, which resulted in a significant increase of miR-375 in MSC exosomes. These exosomes had enhanced abilities of bone regeneration in calvarial defects [72]. The exosomes from the TNF-*α*-primed cells had an elevated level of Wnt-3a compared with unprimed cells, contributing to the enhanced osteoinductive effects of exosomes [73]. HIF-1*α* can stimulate BMSC osteogenic differentiation and enhance angiogenic cell functions. Li constructed HIF-1*α* mutant BMSCs in which HIF-1*α* expresses continuously even under normoxic conditions. They found that exosomes from HIF-1*α* mutant BMSCs (BMSC-ExosMoU) had stronger osteoinductive capacity than those from the wild-type group [74]. Another study reported the exosomes from BMP2-stimulated macrophages integrated to the titanium implants improved the biofunction of the plants by increasing the expression of ALP, BMP2, growth/differentiation factor (GDF)-15, etc. [75]. The therapeutic ability of exosomes can also be improved via loading exosomes with content such as peptides and siRNA by electroporation, which has been studied in disease models of Parkinson's and Alzheimer's [76].

## 6. Challenges for Clinical Application of Exosomes

Despite their great potential in bone regeneration, several challenges existed in the application of MSC-derived exosomes. Among them, the low yield remains to be a major challenge. Several strategies have been explored to maximize yield, including serum starvation and modulating calcium concentration. Nevertheless, those operations could potentially alter the contents and function of exosomes. Another strategy is to increase the supply of MSCs by immortalization. Researchers found that transfection of the c-myc gene provided infinite cell sources for the production of exosomes [77]. However, MYC transformation may give rise to the risks of tumorigenesis, considering that immortalized cells may produce EVs with an altered content or even worse with prooncogenic factors [78, 79]. Therefore, it is important to find a cell source that is efficient in exosomes production. MSCs are the important source of large-scale production of exosomes. Apart from exosomes derived from BMSCs, dental-derived mesenchymal stem cells (D-dMSCs) exosomes may potentially be an excellent or even superior alternative in terms of bone regeneration, particularly the craniomaxillofacial bone. D-dMSCs are abundant, including dental pulp stem cells (DPSCs), gingival mesenchymal stem cells (GMSCs), periodontal ligament stem cells (PDLSCs), dental follicle progenitors (DFPCs), and periapical cyst-mesenchymal stem cells (PCy-MSCs). The procedure of harvesting D-dMSCs is noninvasive. Biological “waste” such as orthodontic teeth, the deciduous teeth, and even the periapical inflammatory cystitis can be the smart source of D-dMSCs. Among the various kinds of D-dMSCs, PCy-MSCs presented a better capability towards osteogenic commitment [80, 81]. Whether the exosomes derived from PCy-MSCs play a better role in bone regeneration remained unexplored. Future studies are needed to clarify in depth the secretomes of the abovementioned MSCs, aiming to figure out the most effective cell source. Besides, when isolating MSCs such as gingival MSCs, new technologies like bioimpedance assay may potentially help identify the healthy and the early potential lesion [82] to ensure harvesting exosomes from healthy cells.

The target specificity of exosomes needs to be further studied and utilized. The internalization of exosomes is realized through cell-exosome interactions involving transmembrane proteins and ECM proteins [83, 84], of which the underlying mechanisms remain unclear. Ligand-receptor recognition may serve a major role in the binding of exosomes to recipient cells. To optimize targeting specificity, antigen or ligands should be developed to attach to the membranes of exosomes. Exosomes loaded with MAGE (melanoma-associated antigen) were used to target the lung cancer cells in a clinical trial (clinicaltrials.gov/NCT01159288). One study used this kind of engineered exosomes to deliver siRNA to the brain. They pretransfected dendritic cells with a plasmid with the neuron-specific RVG peptide clone into the exosomal membrane protein Lamp2b. These exosomes delivered siRNAs specifically to the brain without non-specific delivery and achieved strong silencing of BACE1, a therapeutic target of Alzheimer's disease [85].

The procedures of isolation and administration should be taken into consideration when making conclusions. Important differences occur in terms of the quality and the RNA profiling when using different isolation protocols, such as centrifugation, chromatography, filtration, and polymer-based precipitation [86]. Up to now, there is no consensus on isolation protocols. More standardized methods of preparation should be carried out to get comparable and reliable results.

## 7. Concluding Remarks

The role of exosomes in bone regeneration has been well recognized, and the noncoding RNAs play an important role in exosomes-regulated osteogenic differentiation. If the abovementioned challenges are met, the MSC-derived exosomes for cell-free therapy may offer an elegant alternative for the treatment of bone defects.

## Figures and Tables

**Table 1:** Reported roles of exosomal ncRNAs in osteogenesis.

Origin of exosomes	Experimental objective	Content analysis	Potential targets	In vitro effect	In vivo effect
MSCs	To identify the exosomal microRNA profiles in different osteogenic differentiation stages of MSCs	miR-31, miR-221, and miR-144 were decreased in exosomes from the late stage of osteogenic differentiation; miR-21 was upregulated.	miR-31: Osterix (Osx) and special AT-rich sequence-binding protein 2 (Satb2); miR-221: Runx2; miR-144; Smad4; miR-21: pry1, Sox2, Smad7	Exosomes derived from hMSCs induce osteogenic differentiation of hMSCs in a stage-dependent manner	—
Myoblast	To study the cross-talk between muscle and bone	miR-27a-3p	Apc, a negative regulator of b-catenin; Atf3	Myoblast exosomes affect the osteogenic differentiation of preosteoblasts	
Preosteoblasts	To elucidate the effect of exosomal miRNAs on preosteoblast differentiation	Let-7 miRNA	The high-mobility group AT-hook 2 (Hmga2)	Exosomes whose let-7 was inactivated efficiently lost their osteogenic differentiation capacity.	
Osteoblasts	To study the effect of the exosomes derived from mineralizing osteoblasts on the osteogenic differentiation of MSCs.	miR-667-3p, miR-6769b-5p, miR-7044-5p, miR-7668-3p, and miR-874-3p	Axin1, an important negative regulator of Wnt signaling pathway	Exosomes from MC3T3- E1 under 21 days of osteogenesis induction promote bone marrow stromal cell differentiation to osteoblasts	
BMSCs	To identify the miRNA profile in exosomes derived from aged mice	miR-183 cluster (miR-96/-182/-183)	Heme oxygenase-1 (Hmox1)	Aged exosomes inhibit the osteogenic differentiation of BMSCs	
BMSC	To study the underlying mechanism of MSCT ameliorating osteopenia in Fas-deficient-MRL/lpr mice	miR-29b	Dnmt1	Improved osteogenic differentiation of MSCs from systemic lupus erythematosus (SLE) model	Infusing exosomes rescues osteoporotic phenotype
Adipose mesenchymal stem cells (ASCs)	To investigate whether exosomes derived from miR-375-overexpressing hASCs could enhance bone regeneration	miR-375	Insulin-like growth factor-binding protein 3 (lgfbp3)	Improved the osteogenic differentiation of hBMSCs	Enhanced the bone regeneration in calvarial defect
Multiple myeloma (MM)--MSCs	To understand the myeloma-stroma interactions	RUNX-AS1	Runx2	Exosomes from MM-MSCs impaired the osteogenic differentiation of MSCs	Inhibitor of exosome secretion, prevented bone loss in MM
BMSC	To characterize the full small RNAome of MSC-produced exosomes	5′ halves of tRNA	Runx2, Sox11, Smad3		
